# Transcriptomic analysis of chicken *Myozenin 3* regulation reveals its potential role in cell proliferation

**DOI:** 10.1371/journal.pone.0189476

**Published:** 2017-12-13

**Authors:** Maosen Ye, Fei Ye, Liutao He, Bin Luo, Fuling Yang, Can Cui, Xiaoling Zhao, Huadong Yin, Diyan Li, Hengyong Xu, Yan Wang, Qing Zhu

**Affiliations:** Farm Animal Genetic Resources Exploration and Innovation Key Laboratory of Sichuan Province, Sichuan Agricultural University, Chengdu Campus, Chengdu, China; University of Minnesota Medical Center, UNITED STATES

## Abstract

Embryonic muscle development and fibre type differentiation has always been a topic of great importance due to its impact on both human health and farm animal financial values. *Myozenin3* (*Myoz3*) is an important candidate gene that may regulate these processes. In the current study, we knocked down and overexpressed *Myoz3* in chicken embryonic fibroblasts (CEFs) and chicken myoblasts, then utilized RNA-seq technology to screen genes, pathways and biological processes associated with *Myoz3*. Multiple differentially expressed genes were identified, including *MYH10*, *MYLK2*, *NFAM1*, *MYL4*, *MYL9*, *PDZLIM1*; those can in turn regulate each other and influence the development of muscle fibres. Gene ontology (GO) terms including some involved in positive regulation of cell proliferation were enriched. We further validated our results by testing the activity of cells by cell counting kit-8(CCK-8) and confirmed that under the condition of *Myoz3* overexpression, the proliferation rate of CEFs and myoblasts was significantly upregulated, in addition, expression level of fast muscle specific gene was also significantly upregulated in myoblasts. Pathway enrichment analysis revealed that the PPAR (Peroxisome Proliferator-Activated Receptor) pathway was enriched, suggesting the possibility that *Myoz3* regulates muscle fibre development and differentiation through the PPAR pathway. Our results provide valuable evidence regarding the regulatory functions of *Myoz3* in embryonic cells by screening multiple candidate genes, biological processes and pathways associated with *Myoz3*.

## Introduction

Muscle fibres are highly diverse in colour, contractile properties and metabolic mechanisms. Based on those properties, muscle fibres can be generally divided into 2 categories, namely, white and red muscle fibres. White muscle fibres are characterized by glycolytic metabolism and are specialized for fast and transitory activities. Red muscle fibres are rich in myoglobin and oxidative enzymes and possess the capacity for continuous activity but contract relatively slowly [1, 2]. Muscle fibre type can influence multiple important physiological and pathological properties regarding skeleton and cardiac muscle, including muscle hypertrophy [3, 4], exercise endurance, speed and glucose tolerance [5, 6]. In the field of farm animal research, muscle fibre type is also one of the key factors that influence the meat quality, including meat colour and water holding capacity [7–9]. Different metabolic mechanisms result in different basal metabolic rates, a point of importance for maximizing the feed conversion ratios of farm animals. Myozenin 3 (*Myoz3*) is one of the candidate genes that may influence muscle fibre type.

There are three members in the *Myozenin* (*Myoz*) family, including *Myozenin 1* (*Myoz1*), *Myozenin 2* (*Myoz2*), and *Myozenin 3* (*Myoz3*); they encode the calsarcin-2 (FATZ1), calsarcin-1 (FATZ2) and calsarcin-3 (FATZ3) proteins, respectively. Since its discovery by three independent groups in the period of 2 years, the *Myoz* family has emerged as one of the most intensively studied gene families controlling muscle fibre type. The Z-disc plays important roles in both sarcomere structure and signal transduction and is formed by dozens of proteins, including the *Myoz* family [10–12]. Myoz can interact with multiple Z-disc proteins including α-actinin 2, telethonin, γ-filamin/ABP-L [10], myotilin [13], and calcineurin [11]. In addition, calsarcin-3 is able to interact with the PDZ-LIM domain protein ZASP/Cypher/Oracle [14]. The myozenin family shows a muscle fibre type preference in its expression patterns: while *Myoz2* is mainly expressed in slow-twitch skeletal muscle and cardiac muscle, *Myoz1* and *Myoz3* are predominantly expressed in fast-twitch skeletal muscle.

Studies of the *Myoz* family’s role in muscle fibre type diversity have mainly focused on their negative regulatory effect on calcineurin (CaN) activity by direct binding. Activation of CaN in skeletal myocytes selectively upregulates slow-fibre-specific gene promoters [15] and hence drives a transition from fast to slow muscle fibres. *Myoz2* knockout mice showed inappropriate CaN activity, which resulted in an excess of slow skeletal muscle fibres and enhanced the cardiac growth response to pressure overload [16]. *Myoz1-*deficient mice showed substantially reduced body weight and fast-twitch muscle, and they displayed markedly improved performance and enhanced running distances, also due to aberrant CaN/NFAT overactivation [17]. Inhibition of the CaN signalling pathway by calsarcin-1 can also help prevent cardiomyocyte hypertrophy induced by Ang-II [18]; four residues of calsarcin-1 undergo phosphorylation during pressure overload, resulting in enrichment in cardiac nuclei [19]. In addition, *Myoz2* is a candidate gene for hypertrophic cardiomyopathy (HCM), as established by haplotype mapping of 516 HCM probands [20].

Due to key roles in muscle fibre differentiation, the *Myoz* family is also of great interests among farm animal researchers. Expression profiling of the *Myoz* family in mammals such as swine [21–23] and goats [24] reveal an expression pattern similar to that in mice. An association study also indicates that SNPs of *Myoz* are associated with traits that are influenced by muscle fibre types [25]. However, no research regarding avian *Myoz3* has been reported[26].

The chicken is one of the most financially important birds and also among the most thoroughly studied in terms of genetics, its genome having been published in 2004. Additionally, the chicken is the classic model for the study of vertebrate embryonic development. In the current study, we separately knocked down and overexpressed *Myoz3* in chicken embryonic fibroblasts (CEFs, which are widely used to study chicken embryonic development). Then, we took advantage of the rapid development of high-throughput mRNA sequencing technology to investigate the chicken *Myoz3* gene’s regulatory role at the transcriptome level to identify novel pathways and genes that respond to changes in the expression level of chicken *Myoz3*. We hope to provide evidence to expand our knowledge regarding *Myoz3*.

## Materials and methods

### siRNA synthesis and vector construction

To knock down the expression level of *Myoz3*, three pairs of short interfering RNAs (siRNAs) targeting *Myoz3*’s CDS (coding sequence) and one NC (non-specific control) were designed and synthesized by Sangon Biotech (Table 1) (Shanghai, China). An overexpression vector was constructed by cloning *Myoz3* CDS into the lentivirus vector GM-1013L050 (pLVX-3Flag-MCS-IRES-ZsGreen1), provided by Genomiditech (Shanghai, China).

**Table 1:** siRNA information.

siRNA	Direction	Sequence
*Myoz3*-427	Sense	GGAUGCAGCGCUUUGUCUUTT
	Antisense	AAGACAAAGCGCUGCAUCCTT
*Myoz3*-809	Sense	GCCCAUGAAACUCCCACAUTT
	Antisense	AUGUGGGAGUUUCAUGGGCTT
*Myoz3*-983	Sense	GGUUCUGCCUGAGAGUGAUTT
	Antisense	AUCACUCUCAGGCAGAACCTT
NC (Non-specific control)	Sense	UUCUCCGAACGUGUCACGUTT
	Antisense	ACGUGACACGUUCGGAGAATT

### Cell cultivation, transfection and proliferation test

All the fertilized SPF (specific pathogen free) chicken eggs used in this study were purchased from Meili Breeding Corporation (Beijing, China).

Primary chicken embryonic fibroblasts were obtained from 9-day-old SPF (specific-pathogen-free) chicken embryos. Chicken embryos were first removed of their head and abdominal organs and bones, the remaining tissues were minced and digested with 0.25% trypsin, the suspension were filtered and plated in 12 wells and 96 wells cell culture plates.

Primary chicken myoblasts were isolated as described by Shumao Lin. et. al[27]. In brief, leg muscle was harvested from 11-day-old chicken embryos and minced for further 0.1% collagenase type I (Invitrogen, Carlsbad, CA, USA) digestion. Then the suspension was subjected to a density gradient centrifugation in three discontinuous layers with 20, 30 and 55% Percoll (Solarbio Beijing China), and the cell suspension between the interface of 30 and 55% Percoll was collected and plated in 12 wells and 96 wells cell culture plates.

CEFs and myoblasts were cultured in Dulbecco’s modified Eagle’s medium (Thermo Scientific, USA) with 10% fetal bovine serum (Thermo Scientific, USA) that was filtered with 20 nm filters before use. In addition, 100 μg/mL streptomycin and 100 U/mL penicillin (Thermo Scientific, USA) were added to the cell culture medium. The cells were cultured in 12-wells plates at 37°C in a humified incubator (Thermo Fisher Scientific, USA) with CO_2_ concentration set to 5%. Then, following the manufacturer’s protocol, 20 pmol of each siRNA and NC and 20 μg of expression vector and control vector were transfected into 5×10^5^ CEFs using Lipofectamine 3000 (Invitrogen, USA) in three biological replicates. After 48 hours, RNA was extracted from the transfected cells using TRIzol reagent (Invitrogen, USA). Cell proliferation was tested using a Cell Counting Kit-8 (CCK-8) in the condition of overexpression, following the manufacturer’s instructions. Cells for the proliferation test were cultured in 96-well plates and maintained at 40% of maximum density before transfection.

### RNA-seq and data analysis

Treated CEFs were subjected to RNA-seq, the total RNA was extracted using TRIzol reagent and diluted with RNase-free H_2_O (Tiangen, China). The concentration and purity of the extracted RNA was measured using the NanoDrop 2000 (Thermo Scientific, USA), and RNA degradation and contamination were assessed on 1% agarose gels.

A total of 12 libraries (one library per transfected sample) were constructed, including 3 control samples transfected with control vector, named Control; 3 overexpression samples transfected with overexpression vectors, named Over; 3 knockdown samples transfected with siRNA of *Myoz3*, named Inter; and 3 NC samples transfected with non-specific control siRNA, named NC. A total of 3 μg of RNA per sample was used as input material for preparations. Sequencing libraries were generated using a NEBNext® Ultra™ RNA Library Prep Kit for Illumina (NEB, USA) following the manufacturer’s recommendations, and index codes were added to attribute sequences to each sample. After library preparation, an Illumina HiSeq 4000 was used to generate 120 bp/150 bp paired-end reads. The data files from RNA-seq analysis have been deposited in NCBI's Gene Expression Omnibus, and are accessible through GEO Series accession number GSE99146 (https://www.ncbi.nlm.nih.gov/geo/query/acc.cgi?acc=GSE99146).

The raw data in fastq format were first subjected to the FastQC for quality assessment, then we applyed a in house python script to remove reads containing adapter or poly-N sequences as well as low quality reads and obtain clean reads. Reference genome and gene model annotation files were downloaded from the genome website directly. An index of the reference genome was built using Bowtie v2.2.3 [28], and paired-end clean reads were aligned to the reference genome using TopHat v2.0.12 [29, 30]. HTSeq v0.6.1 was used to count the number of reads mapped to each gene and generate a count table for further analysis [31].

Differentially expressed genes between groups were analysed using edgeR [32], applying LRT (likelihood ratio test) methods, and the P values were adjust using the BH method with a cutoff value of q<0.05. Function annotations for significantly differentially expressed genes were performed using the DAVID website [33]. The enriched gene ontology (GO) terms on biological processes and the pathways obtained from DAVID functional analysis were filtered for significance by gene counts ≥ 3, p-value<0.05. Protein interaction analyses were performed on the STRING website (http://www.string-db.org/).

### Quantitative reverse transcription PCR

For qRT-PCR, first-strand cDNA was synthesized from 1 μg of total RNA using the PrimeScript RT Reagent Kit (Perfect Real-Time) (TaKaRa, Biotechnology Co. Ltd., Dalian, China). The reactions were performed under the following conditions: 42°C for 2 min, 37°C for 15 min and 85°C for 5 s.

qRT-PCR was conducted with two pairs of primers designed with Primer Premier 5 software (Table 2). *GAPDH* was chosen as the housekeeping gene for normalization. An 11 μL reaction containing 6 μL of SYBR premix Ex Taq^TM^ (TaKaRa, Biotechnology Co. Ltd., Dalian, China), 1 μL of cDNA, 0.5 μL of forward primer, 0.5 μL of reverse primer and 3 μL of RNase-free H_2_O (Tiangen, Beijin, China) was used for qRT-PCR. The reactions were carried out with the following amplification conditions: 95°C for 10 s followed by 40 cycles of 95°C for 5 s and 60°C (or the appropriate annealing temperature) for 30s. Each sample was run in 3 technical replicates. The $2^{-\Delta \Delta C_{t}}$ method was applied to quantify mRNA expression levels.

**Table 2:** Primers for qRT-PCR.

Gene	Direction	Sequence	AT (en
*CaN*	Forward	GTTTTCTCCATACAGCTGTCCC	61.2
	Reverse	TCGCAATGCAACGCTTTCTT	
*Myoz3*	Forward	TTCTAGCAGTGACAGGCAGC	60
	Reverse	AGCTTTGTGTTTGCGCTCAG	
*MYH10*	Forward	GATCTGGATCATCAGCGCCA	58.6
	Reverse	GCACGATCTCTCTCCTCTGC	
*MYLK2*	Forward	CTGCACAGGAAGGGAGGAAG	62
	Reverse	GGTGAGCAGCAACACAAAGG	
*MYL4*	Forward	GCGGAGCAGATCGAAGAGTT	61.4
	Reverse	CAGCACCTTCAGGACCTCAG	
*MYL9*	Forward	AACATGTCCAGCAAACGTGC	58.6
	Reverse	AGCGAAGACATTGGAGGTGG	
*NFAM1*	Forward	GCGGAAGGGAGAAACAGACA	58.4
	Reverse	ACTCGATAGGGTTGGAGGCT	
*ITGA8*	Forward	TGTGGGTGCGTTTGGAGCTG	59.4
	Reverse	ACAGGCCACGAAAAGCGGAG	
*GAPDH*	Forward	AGGACCAGGTTGTCTCCTGT	60
	Reverse	CCATCAAGTCCACAACACGG	
*PPM1J*	Forward	GAGAAGGCGGTTTCCCATGA	60.2
	Reverse	CGGATGATAATGGCCCTGCT	
*ECM2*	Forward	AAAGGACGTGCGGACACTTT	60
	Reverse	CTCCTAAGGGCTGCACTTGT	
*OASL*	Forward	CATGAGCCTGACCAGGAAGG	60
	Reverse	AGCAGCACGATGTCGTAGAA	
*FAP*	Forward	GTCGAGTTGGTGTGCAATGG	60
	Reverse	CCTGCCCATCCTGTTTGACT	
*MPRIP*	Forward	ATCCCCTGTGAACACCACTG	60
	Reverse	GCCTTCCTTCAGGCTCTACG	
*PDLIM1*	Forward	TCAGAGGAGAAAGAGGGGTGT	60
	Reverse	GTGACCACCTCGTAGCCTTC	
*PKM*	Forward	AGCAGCAGGAGACACCGAAC	60.2
	Reverse	ATGCCGGTGTTTCTGGCAAT	
*MYHC7B*	Forward	TCAAGCAGCGCTACCGTATT	60
	Reverse	CATCTCTTCCAGCATGCCCA	
*MYH1F*	Forward	ACTTGGTACCACAAGAGCCC	59.6
	Reverse	GCTTGTTCTGGGCCTCAATC	

AT: Annealing temperature

## Results

### Knockdown and overexpression of *Myoz3* in CEFs and myoblasts

First, we thought to determine whether Myoz3 is expressed in CEFs and to which level is Myoz3 expressed in CEFs compare to myoblasts and adult breast muscles (tissue from our previously publication [34]). Our results suggest that *Myoz3* is expressed in CEFs, although it’s significantly lower than in breast muscles and myoblasts (about 4 and 2 times, respectively). Our results suggest that *Myoz3* play a role in chicken embryonic fibroblasts.

**Fig 1:**
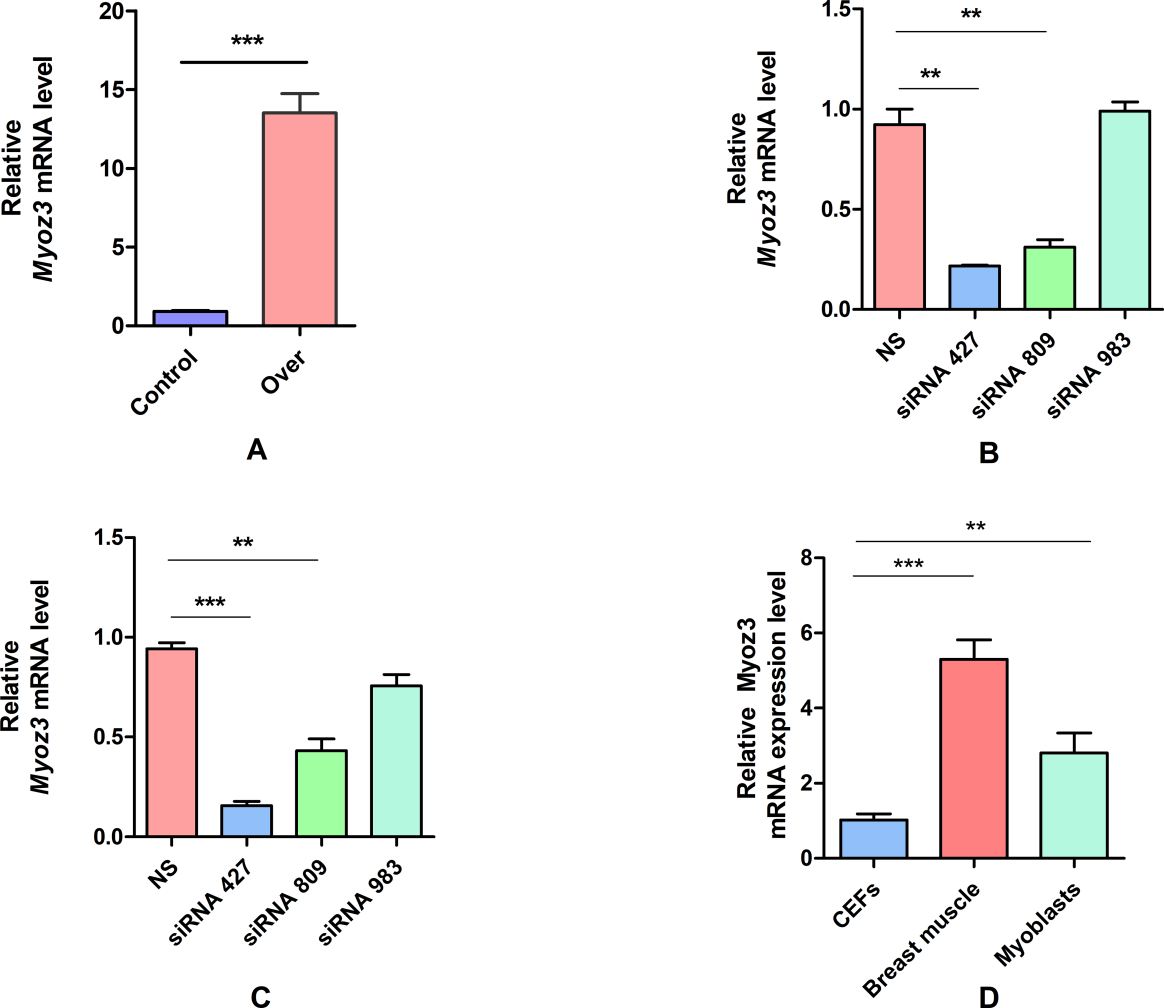
*Myoz3* siRNA and overexpression vector screening. (A) *Myoz3* expression level in breast muscle, CEFs and Myoblasts. (B) *Myoz3* relative expression level in CEFs after transfection with non-specific siRNA (Control), and candidate siRNA. (C) Myoz3 relative expression level in myoblasts after transfection with non-specific siRNA (Control), and candidate siRNA. (D) *Myoz3* relative expression level in CEFs after transfection with empty vector and overexpression vector (Over). The error bars represent SEM; all experiments were replicated three times. *P<0.05, **P<0.01, ***P<0.001.

To analyse the function of *Myoz3 in vitro*, we knocked down and overexpressed the *Myoz3* gene in CEFs and myoblasts by transfecting them with siRNA and overexpression vectors, respectively. First, after we tested the efficiency of various siRNAs, siRNA 427 was identified as the most efficient siRNA for use in further experiments because of its knockdown efficiency of over 20% compared with NS (non-specific control). The overexpression vector of *Myoz3* was driven by the CMV promotor, and 24 hours after transfection, the expression level was elevated more than 10-fold compared with cells that were transfected with empty vectors (Fig 1).

### RNA sequencing

A total of 12 libraries were prepared. Those libraries were Inter (CEF transfected with siRNA), NC (CEF transfected with non-specific RNA), Over (cell transfected with overexpression vector), and Control (cell transfected with empty vector), each of them prepared in 3 biological replicates. An Illumina Hi-Seq 4000 was used to generate paired-end reads. An average 46.77 million reads were generated for all the libraries, with Q20 more than 97.3% and Q30 more than 93.4% (S1 Table). More than 80% of percent reads were mapped to the galGal5 chicken genome (S2 Table) by TopHat2. The data files from the RNA-seq analysis have been deposited in the NCBI’s Gene Expression Omnibus.

### Differentially expressed genes and qRT-PCR validation

**Fig 2:**
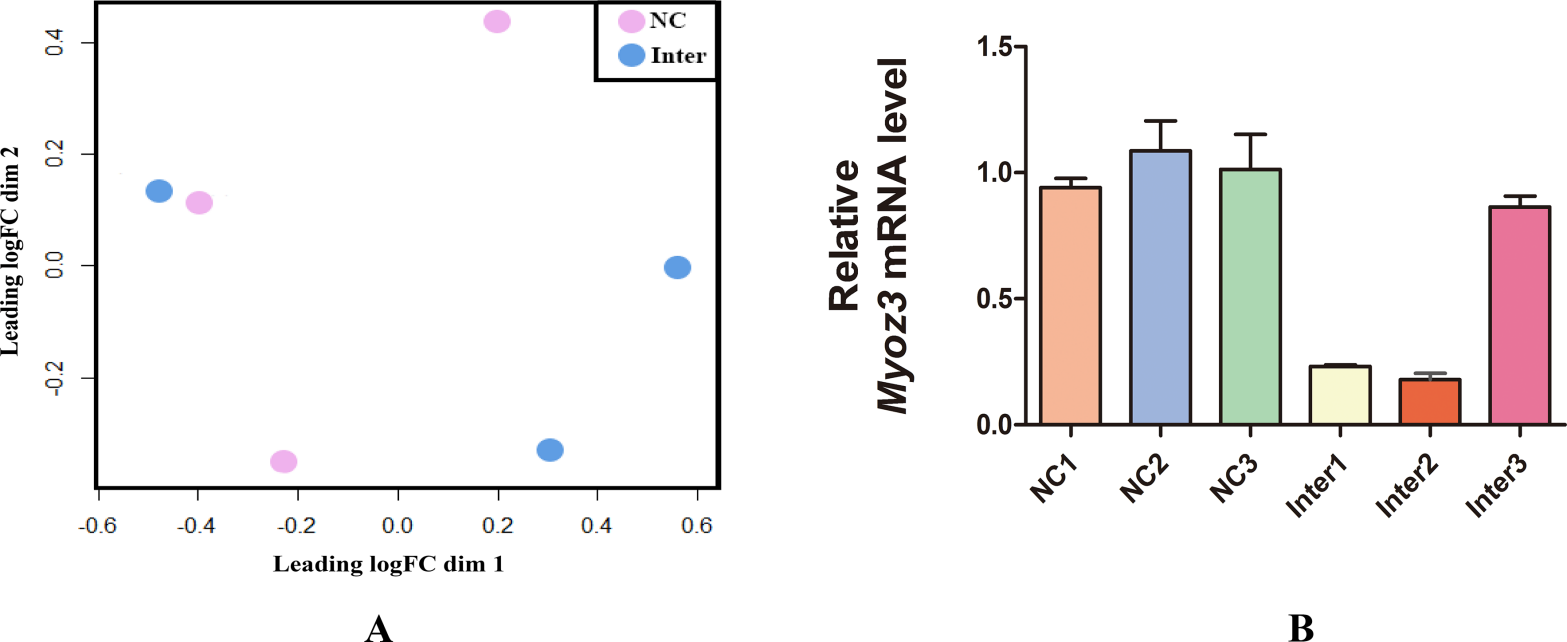
The 3^rd^ sample of Inter is abnormal due to inefficient knockdown. **(A)** An MDS plot of NC and Inter illustrates the abnormality of the 3^rd^ Inter sample. **(B)** The expression level was not sufficiently knocked down in the 3^rd^ Inter group. The genes that were differentially expressed between the two experiment conditions are presented. The error bars indicate SEM for three replicates (qRT-PCR). Significance was not calculated because to data for each bar do not represent biological replicates.

A count table containing each the ID and read counts of each gene was generated from aligned reads by HTSeq v0.6.1[35]. In addition, the R package edgeR was applied for differentially expressed gene analysis. First, we generated a multidimensional scaling (MDS) plot to evaluate the leading biological coefficient of variation (BCV). We found that the 3^rd^ replicate of Inter was abnormally far from other Inter samples and close to the NC samples; we suspected that the interference was not sufficient, and so we tested the *Myoz3* expression level by qRT-PCR, revealing that the expression level of *Myoz3* in the 3^rd^ Inter group was similar to that of NC (Fig 2). Therefore, we excluded the 3^rd^ NC group from further analysis. The cutoff value for differential expression was an adjusted P<0.05.

**Fig 3:**
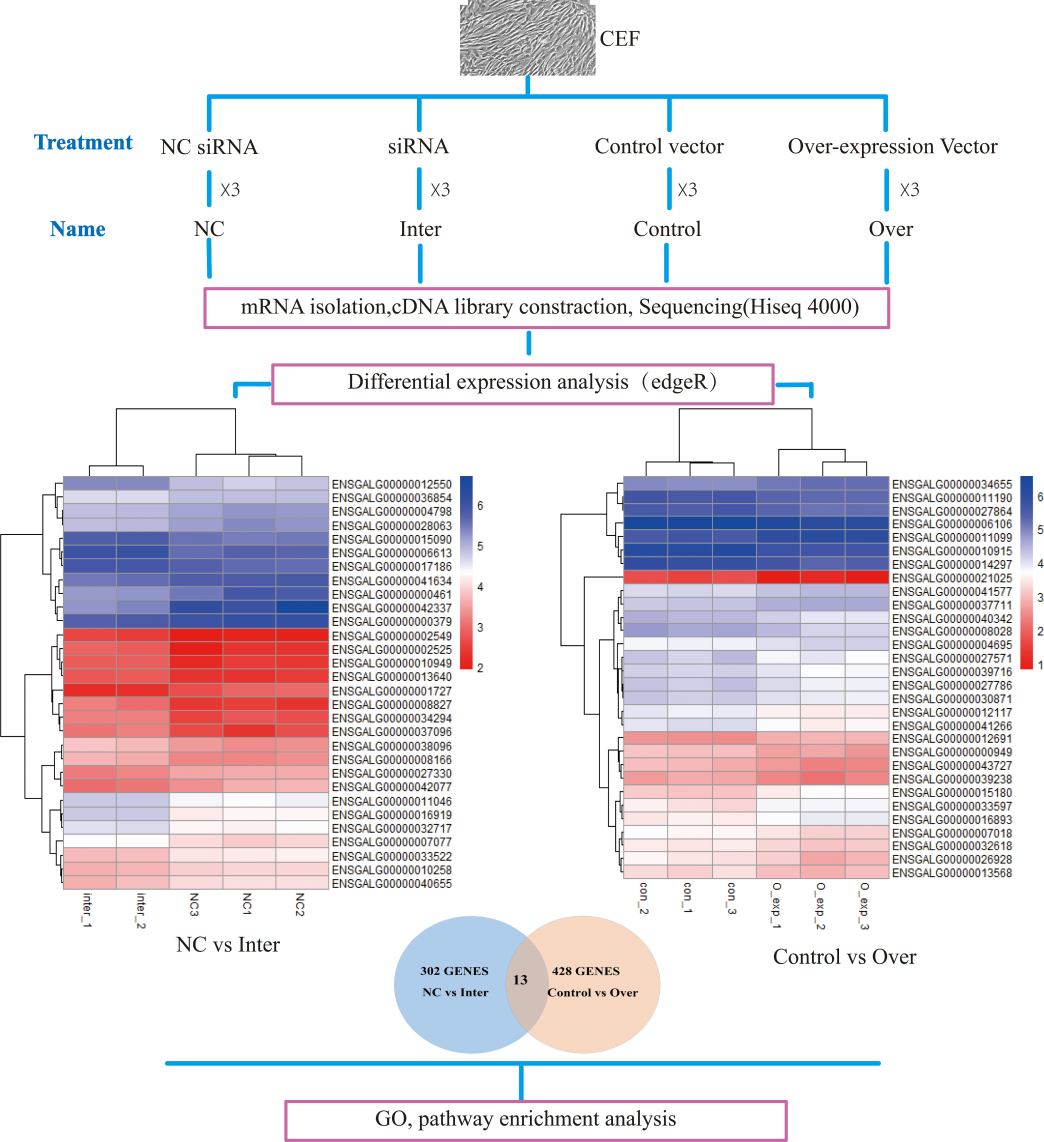
Experimental design and overall results. The expression levels of the top 30 differentially expressed genes are shown in log CPM (copies per million). NC (non-specific control).

When Inter was compared with NC, 302 genes were found to be differentially expressed, of which 84 were significantly downregulated and 226 of them were significantly upregulated, including *MYL4* (myosin light chain 4) and *MYL9* (myosin light chain 9). When Over was compared with Control, 301 genes were downregulated and 127 genes were upregulated. Some genes involved in muscle development including *MYH10* (myosin heavy chain 10) and *MYLK2* (myosin light chain kinase 2) were among the differentially expressed genes. Thirteen genes were differentially expressed in both knockdown and overexpression conditions; they are *VWA5B2* (von Willebrand factor A domain-containing 5B2), SQSTM1 (sequestosome 1), *ASPN* (asporin), *ITGA8* (integrin subunit alpha 8), *PLK3* (Polo-like kinase 3), CRLF1 (cytokine receptor like factor 1), LRP3(LDL receptor related protein 3), TNFRSF6B (TNF receptor superfamily member 6b), THBS (thrombospondin-2 precursor), ENSGALG00000035656, ENSGALG00000028466, ENSGALG00000037711, and ENSGALG00000011668 (Fig 3).

**Fig 4:**
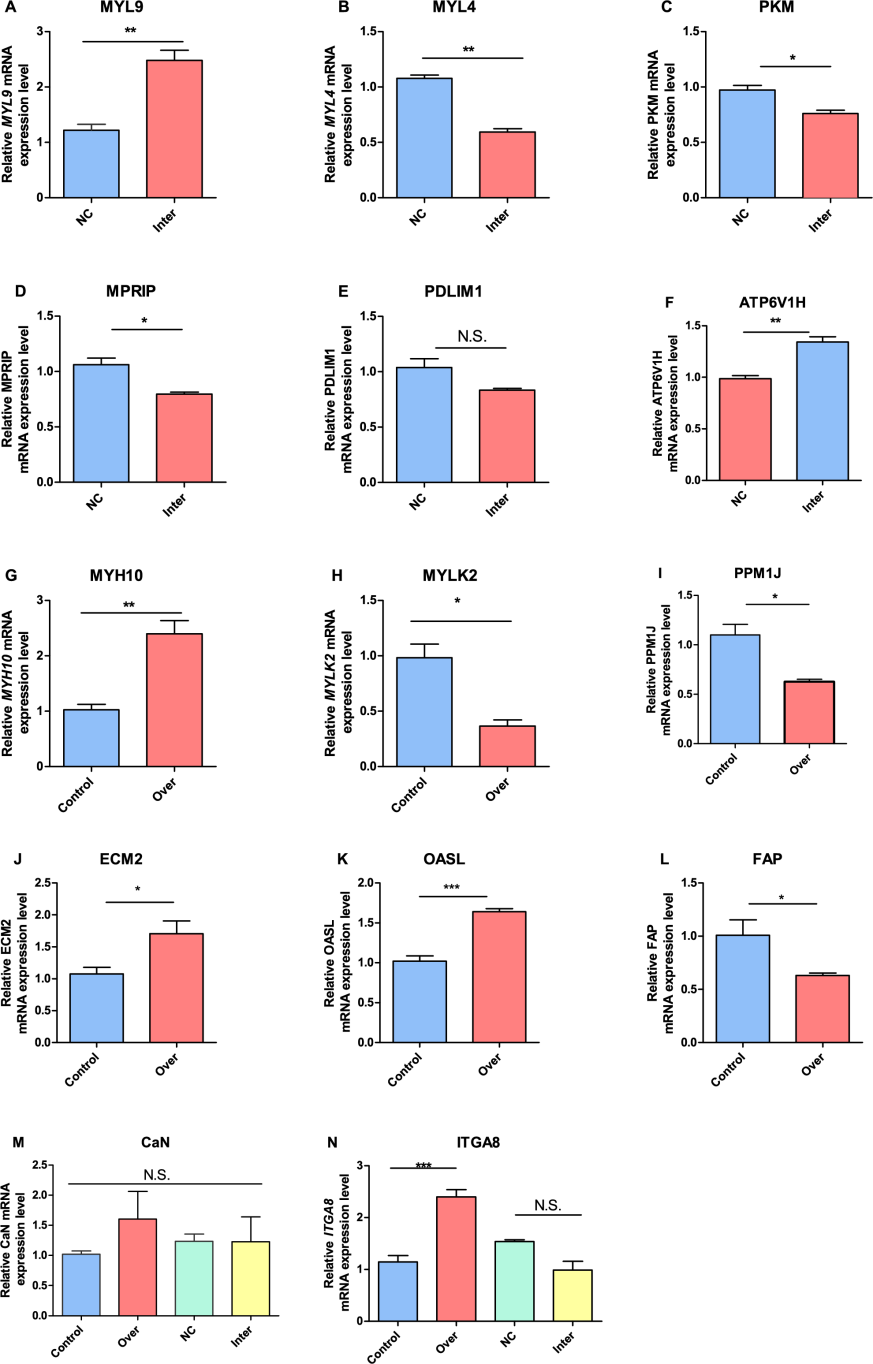
Validation of differentially expressed genes by qRT-PCR. The relative expression level of data was presented by bar plots with error bars represent SEM. All experiments were replicated three times. (A) *MYL9*, (B) *MYL4* (C) *PKM*, (D) *MPRIP*, (E) PDLIM1, (F) *MYH10*, (G) *MYLK2*, (H) *PPM1J*, (I) *ECM2*, (J) *OASL*, (K) *FAP*, (L) *CaN*, (M) *ITGA8*. *P<0.05, **P<0.01, ***P<0.001, N.S. means not significant.

To validate the results that were obtained by RNA-seq, qRT-PCR was performed for each of the 12 libraries. We confirm that except for the 3^rd^ library of Inter, *Myoz3* was expressed as intended in other groups, that is, highly expressed in the Over group and scarcely expressed in the Inter group. Then, we validated genes that were identified as differentially expressed by RNA-seq, including *MYH10*, *MYLK2*, *MYL4*, *MYL9*, ASPN and KLP3, protein phosphatase, Mg2+/Mn2+ dependent, 1J (*PPM1J*), extracellular matrix protein *2* (*ECM2*), 2'-5'-oligoadenylate synthetase-like (*OASL*), fibroblast activation protein, alpha (*FAP*), PDZ and LIM domain 1 (*PDLIM1*), *myosin phosphatase Rho interacting protein* (*MPRIP)*, pyruvate kinase, muscle *(PKM)*, ATPase H+ transporting V1 subunit H (*ATP6V1*). Of 12 genes that were selected as genes to verify (CaN was chosen as a negative control because it has not been identified as a differentially expressed gene), *ITGA8* and *PDLM1* were not statistically correspond to our RNA-seq results, but trends in *ITGA8* and *PDLM1* expression were along similar lines. In addition, all the other results are able to verify our results of differentially expressed gene analysis (Fig 4).

### Gene ontology (GO), pathway and protein interaction analyses

To better understand the differentially expressed genes we filtered from background, we conduct gene ontology and pathway enrichment analysis. The differentially expressed gene lists for both comparisons were submitted to DAVID (http://david.abcc.ncifcrf.gov/) for enriched functional terms in biological processes and pathways.

**Fig 5:**
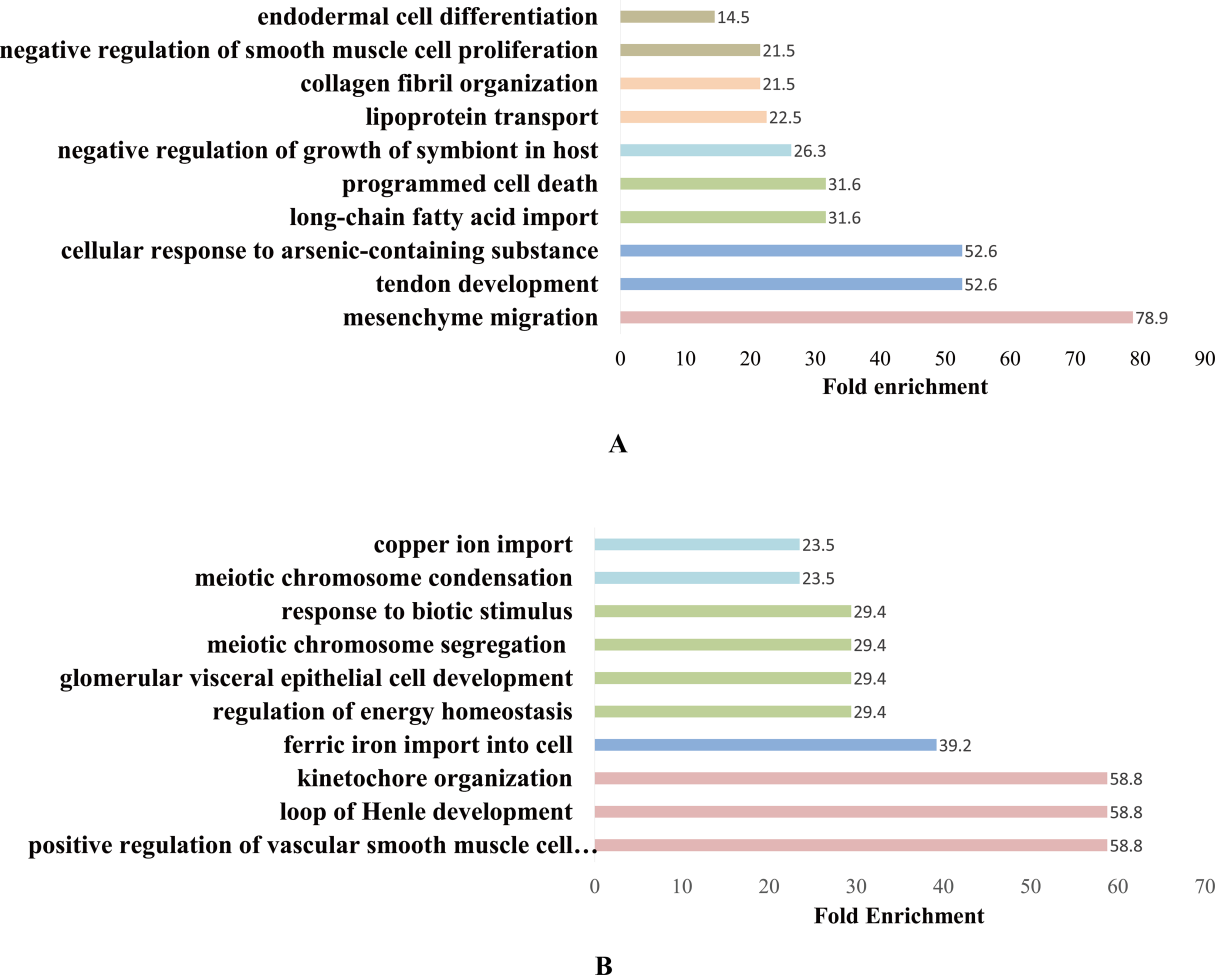
Enriched biological process (BP) terms. (A) BP terms enriched under the knockdown condition (NC vs Inter). (B) BP terms enriched under the overexpression condition (Control vs Over).

Under knockdown condition, pathways such as cardiac muscle contraction, ECM-receptor interaction, PPAR, vascular smooth muscle contraction, and focal adhesion were enriched (S1 Fig). Key words such as calcium and muscle protein were also enriched. Biological process such as mesenchyme migration, tendon development, long-chain fatty acid import was enriched (Fig 5).

Under the overexpression condition, biological process terms such as positive regulation of vascular smooth muscle cell proliferation, loop of Henle development, regulation of energy homeostasis were enriched. Four pathways were found to be enriched, namely, the ErbB signalling pathway, influenza A, herpes simplex infection, and focal adhesion.

**Fig 6:**
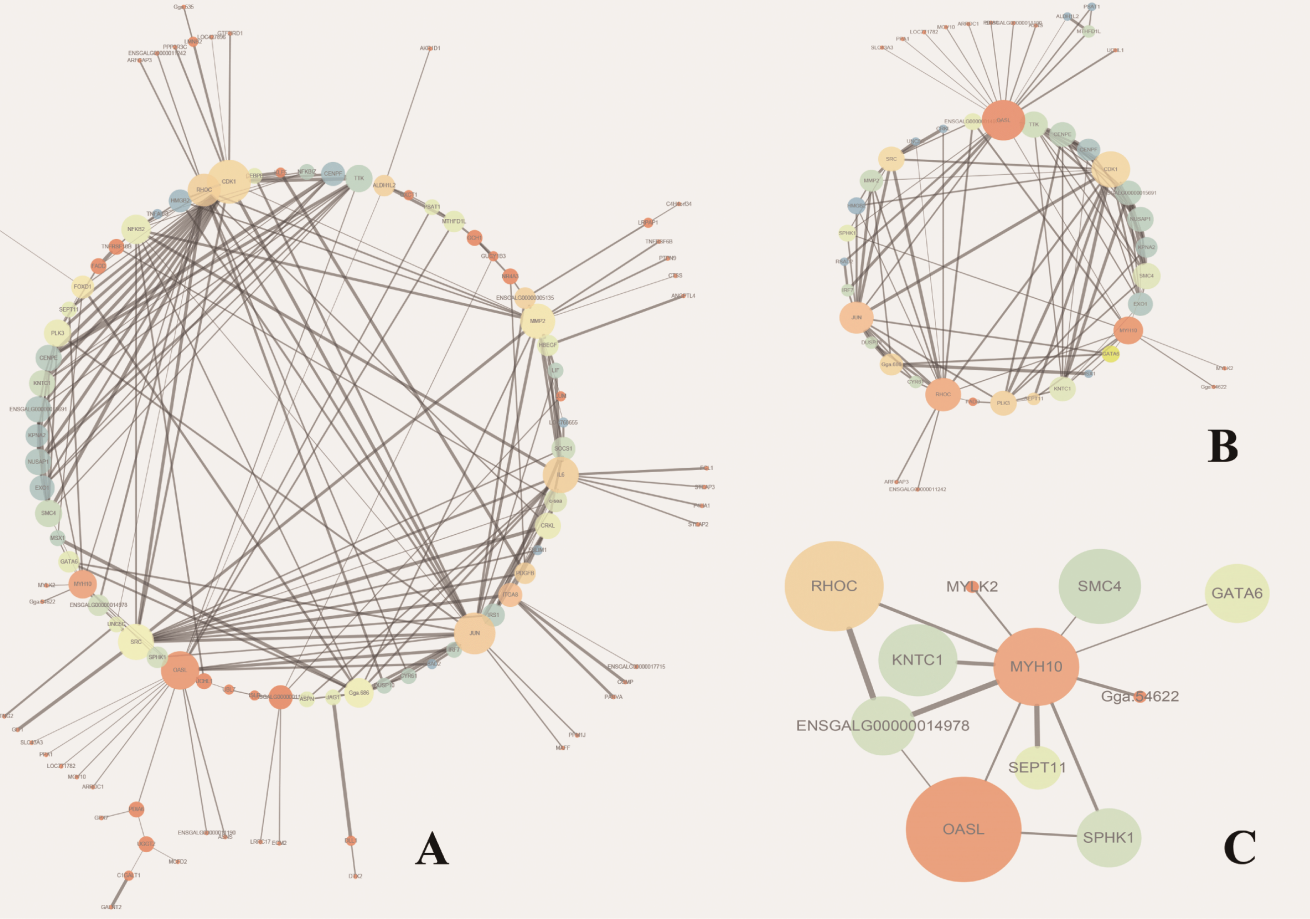
Protein interaction networks under the overexpression condition. (A) Biggest network under the overexpression condition, containing 108 genes. (B) Secondary interaction networks of MYH10, containing 47 genes. (C) Network of genes that directly interact with MYH10, containing 11 genes.

To further illustrate the regulation network among differentially expressed genes, we conducted proteins interaction network analysis. In the overexpression condition, we focused on the interaction network that is centred on MYH10 (Fig 6). MYH10 can directly interact with MYLK2, GATA6, SMC4, and others. The secondary network, including proteins that interact with proteins that interact with MYH10 in differentially expressed genes, contains 47 genes. In the knockdown condition, few interesting networks were identified.

### Cell proliferation rates was elevated under *Myoz3* overexpression condition

**Fig 7:**
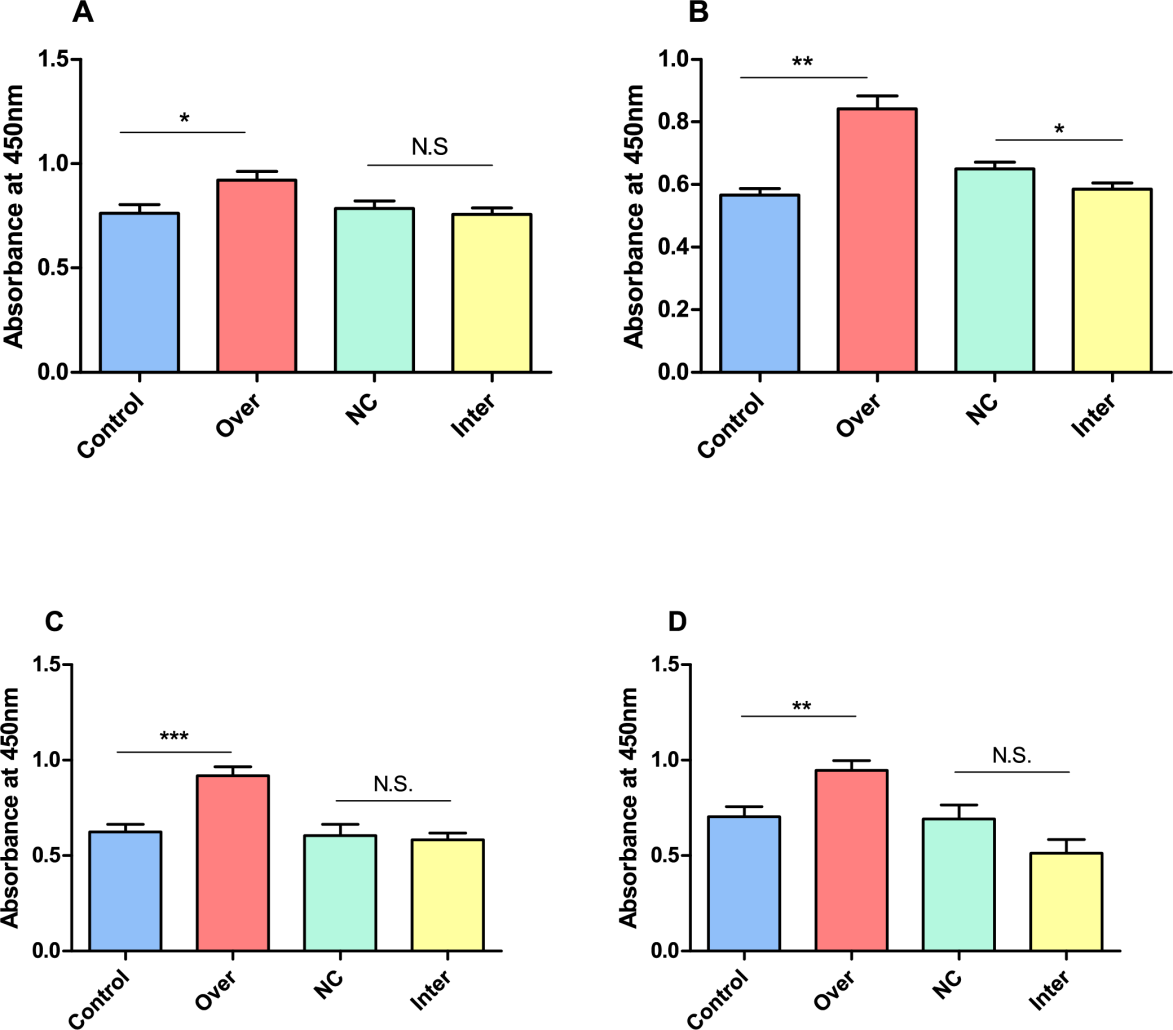
Proliferation by CCK-8 under both *Myoz3* knockdown and overexpression conditions. (A) 24 hours after CEFs transfected with empty vector (Control) or overexpression vector, non-specific siRNA (NC) or *Myoz3* siRNA (Inter). (B) 48 hours after CEFs transfected with empty vector (Control) or overexpression vector, non-specific siRNA (NC) or *Myoz3* siRNA (Inter). (C) 24 hours after Myoblast transfected with empty vector (Control) or overexpression vector, non-specific siRNA (NC) or *Myoz3* siRNA (Inter). (D) 48 hours after myoblasts transfected with empty vector (Control) or overexpression vector, non-specific siRNA (NC) or *Myoz3* siRNA (Inter).

To further support our results from RNA-seq, we conducted a cell proliferation test in both CEFs and myoblasts, since pathways and biological process involve in cell proliferation were enriched, such as the PPAR pathway as well as biological process terms such as positive regulation of vascular smooth muscle cell (VSMC) proliferation. The results show that when *Myoz3* is overexpressed in CEFs and myoblasts, cell proliferation was significantly upregulated, Knockdown of *Myoz3* in both cells results in trend of proliferation inhibition. (Fig 7).

### The fast muscle specific gene was upregulated upon Myoz3 overexpression

**Fig 8:**
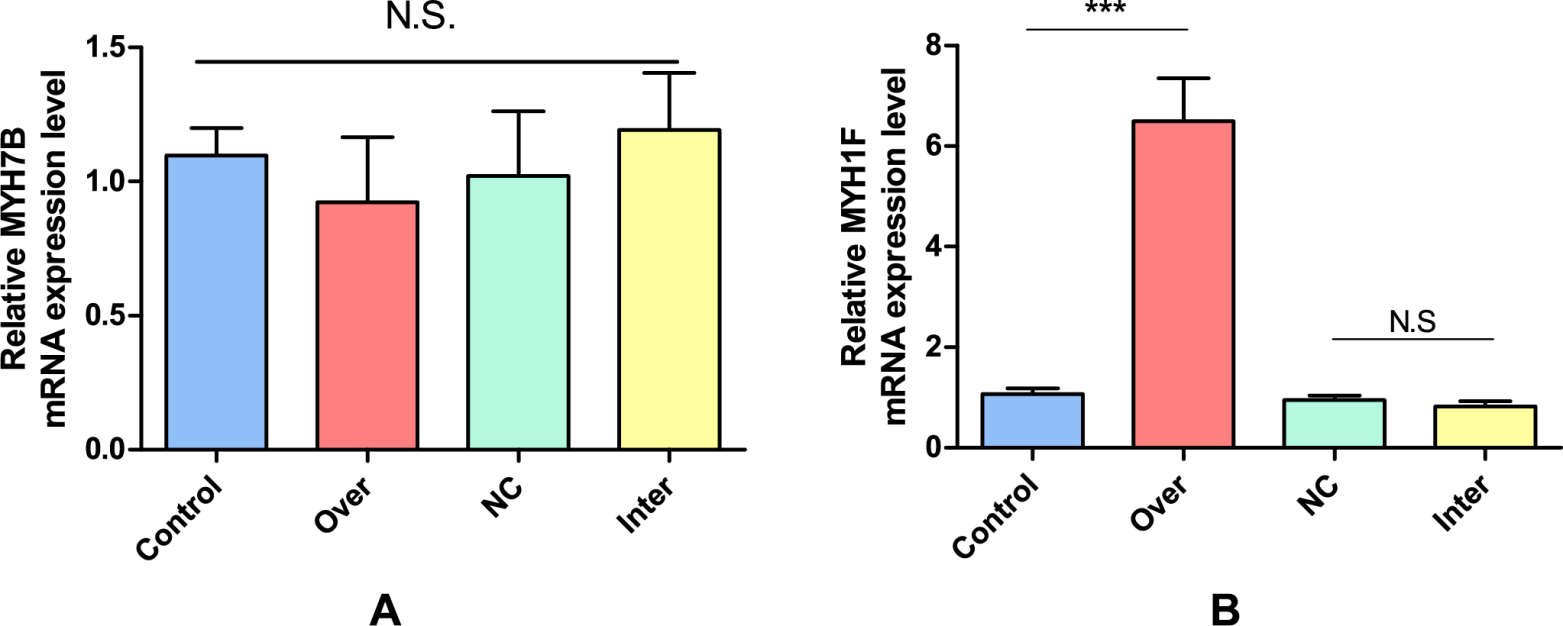
Myoz3 overexpression results in fast muscle specific gene up-regulated but not slow muscle specific gene. (A) Slow muscle specific gene *MYH7B*. (B) Fast muscle specific gene *MYH1F*. NC; non-specific control siRNA. Inter; *Myoz3* siRNA. Control; empty vector. Over; *Myoz3* overexpression vector. The relative expression level of data was presented by bar plots with error bars represent SEM. All experiments were replicated three times. Error bars represent SEM. *P<0.05, **P<0.01, ***P<0.001.

To test whether muscle fibre type specific gene can be regulated by *Myoz3*, we detected the expression level of *MYH7B* (myosin heavy chain 7b), which is slow muscle marker gene; *MYH1F* (myosin heavy chain 1f), which is a fast muscle specific gene. Our results show that in myoblast, overexpression of *Myoz3* gene can lead to *MYH1F* upregulated but not *MYH7B* down regulated. However, no change was observed in *Myoz3* knockdown group (Fig 8) and all CEFs groups (data not shown).

## Discussion

Muscle fibre type differentiation is a delicate process that is regulated both during and after the embryonic period [36, 37]. For animals that have limited activity space after birth/hatching and whose muscle tissue is of great economical value, such as chickens, embryonic development of muscle fibres is a factor of great importance, especially developmental control of muscle fibre types. In this study, we focus on *Myoz3*, a candidate gene for muscle fibre type differentiation, hoping to understand its regulatory roles in embryonic muscle fibre development. We apply the fast-growing technology of deep sequencing to CEFs under both *Myoz3* knockdown and overexpression conditions, hoping to identify pathways and biological process associated with *Myoz3* expression level.

CEFs is one of the most broadly used cell types for studying the embryonic development of chickens [38, 39], and a model for study both chicken signalling pathways [40, 41]. *Myoz3* expression was detected in primary CEFs, however, little is known about *Myoz* family’s role in fibroblasts. we suspect that Myoz3 play a role in CEFs differentiation, and chicken embryonic development. In addition, previous evident suggest that activation of muscle specific gene in non-muscle tissue by forced expression of muscle specific regulator [42]. Therefore, both CEFs and myoblasts were utilized in this study.

Even though little evidence of *Myoz3*’s role in muscle fibre type differentiation has been reported, studies regarding other *Myoz* family proteins can provide valuable information about *Myoz3* function. In human, mice and chickens alike, each member of the Myoz family possesses only one domain, calsarcin. *Myoz2*^-/-^ mice showed an excess of slow-twitch skeletal muscle fibre due to calcineurin activity upregulation, and a foetal gene program was activated in *Myoz2* deficient hearts that caused the Z-discs to become ‘fuzzy’ [16]. *Myoz1*^-/-^ mice showed a reduction in body weight and fast-twitch muscle mass; also, their endurance capacity was increased due to a fibre type shift towards slow-twitch oxidative fibres. Similar to *Myoz2*^-/-^ mice, *Myoz1*^-/-^ mice also showed an increase in Calcineurin/NFAT activity [17], despite the different expression pattern between *Myoz2* and *Myoz1*. In the current study, we identified multiple genes and pathways that are possibly regulated by chicken *Myoz3* in CEFs.

In the overexpression condition, a total of 428 genes were identified as significantly differentially expressed when filtered by the least ratio test (LRT) method applying edgeR, including *MYH10*, which is significantly upregulated, and *MYLK2*, which is significantly downregulated. *MYH10* (*non-muscle myosin II-B*) belong to a non-muscle myosin family that has roles in cell migration, adhesion [43], and polarity as well as cardiac and brain development [44]. It was proposed that the first step of myofibril assembly is the formation of integrin-dependent cell–matrix adhesion, in which MYH10 play a key role, *MYH10* transgenic mutant mice show defects in their heart sarcomeres [45]. *MYH10* can also respond to the Ca^+^-calmodulin pathway, which can activate MYLK (myosin light chain kinase), which then phosphorylates the RLCs (regulatory light chains) of MYH10, which can facilitate myosin interaction with actin filaments, leading to great increases in the Mg^2+^-ATPase activity of myosin in the presence of actin [46]. The *MYLK2* reduction and MYH10 elevation under the *Myoz3* overexpression condition may lead to *MYH10* hypophosphorylation when the Ca^+^-calmodulin pathway is activated. Hence, there is a possibility that *Myoz3* is a negative regulator that inhibit another downstream signal molecular of the Ca^+^ signalling pathway. However, further studies are required regarding how *Myoz3* might regulate *MYLK2*. In addition to phosphorylating MYH10 protein, MYLK2 protein also modulates a variety of contractile processes, including smooth muscle contraction and proliferation [47], so we suspect that *MYLK2* is a key components in *Myoz3* regulatory network.

Under the knockdown condition, 302 genes were differentially expressed, including *MYL9* (myosin light chain 9) and *MYL4* (myosin light chain 4), *PDLIM* (PDZ and LIM domain 1), *PKM* (pyruvate kinase, muscle), *NFAM1* (NFAT activating protein with ITAM motif 1). Myosins are a superfamily of molecular motors that depend on action and are implicated in contraction, cell shape, migration, adhesion, and intracellular transport [48]. *MYL9* is reported to be associated with injury and ageing [49], and *MYL4* is an atrial-specific myosin light chain gene. Mutation of *MYL4* can lead to atrial fibrillation in humans, and *MYL4* mutant zebrafish displayed disruption of sarcomeric structure and atrial enlargement [50]. In the current study, *Myoz3* knockdown results in *MYL4* upregulation, consistent with the fact that *Myoz3* is predominantly expressed in fast-twitch muscle [14], which is not localized in the heart. We suspect that a fast-twitch muscle-specific gene such as *Myoz3* can play a key role in embryonic cell differentiation. *NFAM1* (NFAT activating protein with ITAM motif 1), screened with NFAT-GFP reporter cells for its activating effect on transcription factor NFAT [51], was reported a decade ago and found to be up regulated under *Myoz3* knockdown conditions. Consider that calsarcin-3 can bind with calcineurin and inhibit the activation of NFAT, we suspect that part of the inhibitory role is to negatively regulate the expression level of *NFAM1*. Furthermore, *PDLIM1*, known to interact with *Myoz3*, was downregulated. Both PDLIM1 and Myoz3 are localized in the Z-disc of skeletal muscle, and both contribute to Z-disc formation[52, 53]; therefore, downregulation of both genes may result in aberrant Z-disc signal transduction[54], hence the impact on the differentiation of muscle fibres in the embryonic period.

To better understand the function of *Myoz3*, we conducted gene ontology(GO) analysis. Under *Myoz3* overexpression conditions, terms including positive regulation of vascular smooth muscle cell proliferation were enriched. Under *Myoz3* knockdown conditions, on the other hand, the BP term negative regulation of smooth muscle cell proliferation was enriched, so we test whether a change in the expression of *Myoz3* can influence the cell proliferation of CEFs and myoblasts. As described above, under overexpression conditions, both myoblasts and CEFs had significantly higher activity, confirming our results from RNA-seq data. Pathways were also enriched: under knockdown condition, pathways such as the PPAR signalling pathway and ECM-receptor interaction were enriched. Several prior publications regarding PPAR pathway show that three isoforms of *PPAR* subfamily play a roles in skeletal muscle metabolisms and plasticity, including *PPAR*α, *PPAR*β/δ, *PPAR*γ [55]. The expression level of *PPARα* reflects differences in type Ⅰ muscle fibres associated with pathologically and physiologically induced skeletal muscle fibre type differences [56], and vascular smooth muscle cell proliferation can be inhibited by *PPARα* through suppression of telomerase activity [57]. *PPARα-*overexpressing transgenic mice showed upregulated expression of genes involved in oxidation in skeletal muscle [58]. Regarding *PPARβ/δ*, activation of *PPARδ* in skeleton muscle leads to leads to muscle fibre type transformation, from type II to type I [59], the same study also show that activation of *PPARβ* can also prevent obesity as a results of metabolism alteration. Another study supported these results, Luquet *et*. *al*. show that muscle-specific overexpression of *PPARβ/δ* in mice enhances muscle metabolism (fatty acid flux and b-oxidation) and altered muscle fibre type to increase oxidative type 2a. These mice also show decreased body fat mass and smaller fat cells[60] Furthermore, *PPARβ/δ* activation that was induced by agonist resulted in enhances fatty acid oxidation in skeletal muscle cells [61]. C2C12 myotube was enriched upon *PPARβ* activation along with enhanced mitochondrial biogenesis [62]. In general, PPAR pathway that was enriched under *Myoz3* knockdown condition is highly likely to relate to *Myoz3* regulation, but further study regarding the mechanism as how Myoz3 regulation works require further study.

Although no specific pathway that relates to muscle fibre was found under *Myoz3* overexpression conditions, several muscle development genes were nonetheless found, probably due to the background noise. The pathways and biological processes we identified included not only skeletal muscle regulation but also smooth muscle regulation, so we suspect that *Myoz3* also functions in smooth muscle development in the embryonic period. Furthermore, overexpression of *Myoz3* in myoblasts leads to significantly increase of fast-muscle specific gene expression, which make us believe that chicken gene/pathway annotation may need further improvement.

Another way to better understand and evaluate the pathway formed by differentially expressed gene is to construct protein-protein interaction (PPI) network, our results show that differentially expressed genes encode a highly interconnected network. And further confirmed the results that MYH10 and MYLK2 interact with each other is highly likely molecular process that under *Myoz3* regulation. In addition, GATA6 can also bind to MYH10, which is another possible interaction that explain the function of *Myoz3*. GATA6 is a transcriptional factor that regulate cardiomyocyte hypertrophy[63], smooth muscle contraction [64]. However, PPI construction can only provide us indirect evidence, further experiments regarding protein interaction including CoIP (Co-Immunoprecipitation) and GST pull-down.

In general, by using highly advanced deep sequencing technology for cells under both overexpression and knockdown conditions, we were able to reveal the function of chicken *Myoz3* in embryonic development. To the best of our knowledge, this is the first transcriptomic study applying RNA sequencing technology to study the function of *Myoz3* in any species. Our results provide more than 302 candidate genes regulated by *Myoz3* under *Myoz3* knockdown conditions and 428 candidate genes under *Myoz3*’s regulation under overexpression conditions. Our results indicate that *Myoz3* has the potential to regulate multiple myosin light chain family members, such as *MYL4*, *MYL9*. Non-muscle myosin heavy chain *MYH10* was also found to be regulated by *Myoz3*, as was *MYLK2*, the kinase that phosphorylates *MYH10*. Pathways were also identified. The PPAR pathway is a very promising pathway that is likely involved in *Myoz3*-mediated embryonic development and muscle fibre type differentiation. We also confirmed our results by testing the proliferation of CEFs and myoblasts. Furthermore, muscle fibre type specific gene can also be regulated in myoblast upon *Myoz3* expression alteration. However, due to the knockdown mechanism, our results may contain some background noise from the siRNA transfection and the remaining *Myoz3* that we were unable to be knock down completely; a CRISPR/Cas9-mediated gene knockout method may help us better understand the role of *Myoz3* at a cellular and organism level. Important question such as how chicken *Myoz3* is regulated and the precise mechanism of how chicken *Myoz3* is involved in cell proliferation still need to be answered by further studies.

## Supporting information

S1 Fig Enriched pathways. (A) Pathway enriched under the knockdown condition (NC vs Inter). (B) Pathway enriched under the overexpression condition (Control vs Over).
(TIF)

S1 Table Quality control Raw data and Quality control information. (XLSX)

S2 Table Reads mapping. Reads mapping information.
(XLSX)

S3 Table Inter DE. Differentially expressed gene ID under knockdown condition.
(XLSX)

S4 Table Over DE. Differentially expressed gene ID under overexpression condition.
(XLSX)

S5 Table Counts table. Counts table that generated by HTseq.
(XLSX)
