# Supplementary figures and images for: Transcriptomic analysis of chicken Myozenin 3 regulation reveals its potential role in cell proliferation
Source: PLoS One. 2017 Dec 13;12(12):e0189476. doi: 10.1371/journal.pone.0189476 (PMC5728575; doi:10.1371/journal.pone.0189476)

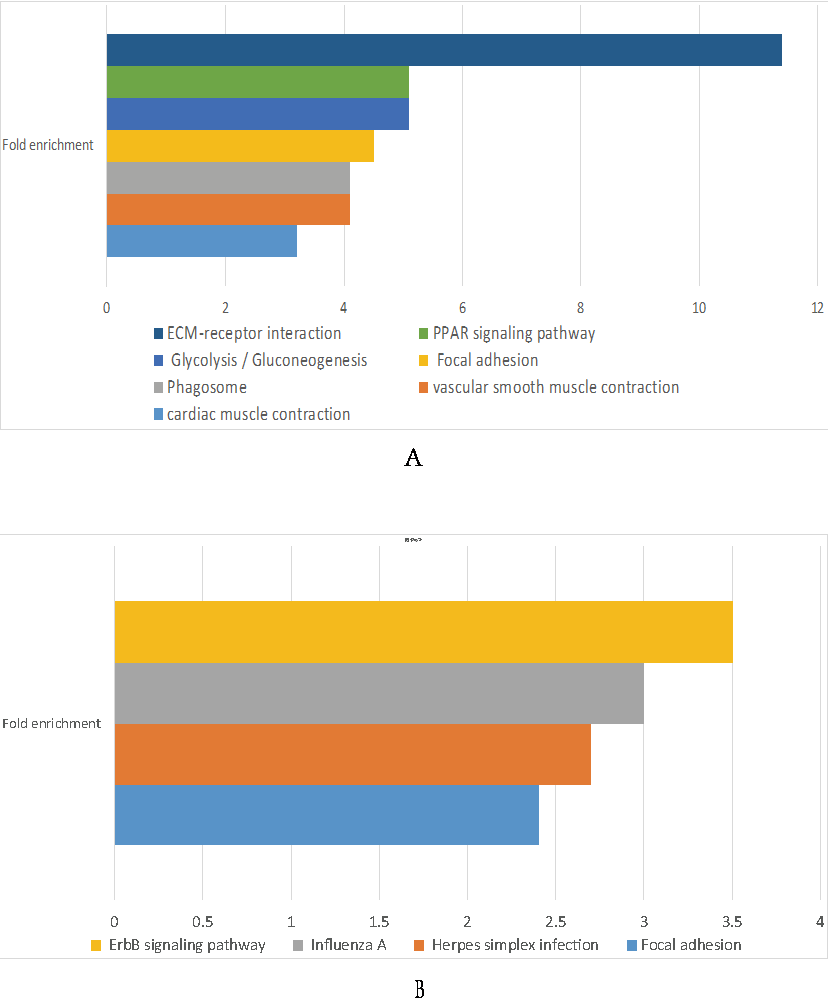

Supplement: S1 Fig — (A) Pathway enriched under the knockdown condition (NC vs Inter). (B) Pathway enriched under the overexpression condition (Control vs Over). (TIF) [file pone.0189476.s001.tif]
